# Comparison of targeted next-generation sequencing and conventional tests for pathogen detection in community-acquired and severe community-acquired pneumonia: a retrospective cohort study

**DOI:** 10.3389/fmicb.2026.1799720

**Published:** 2026-05-11

**Authors:** Feng Wang, Axiang Han, Wanting Hong, Ying Mao, Weijie Sun, Yixuan Hu, Lingling Lu

**Affiliations:** 1 Department of Clinical Laboratory, The First Affiliated Hospital of Ningbo University, Ningbo, Zhejiang, China; 2 Infection Technology Platform, Dian Diagnostics Group Co., Ltd., Hangzhou, China

**Keywords:** clinical diagnostic consistency, community-acquired pneumonia, conventional tests, severe community-acquired pneumonia, targeted next-generation sequencing

## Abstract

**Background:**

Community-acquired pneumonia (CAP) and severe community-acquired pneumonia (sCAP) impose a significant burden on the healthcare system. As a more severe form of CAP, sCAP is associated with a higher mortality rate, and patients frequently require intensive care. Identifying clinical biomarkers to distinguish CAP from sCAP is expected to improve pneumonia management and patient outcomes. Targeted next-generation sequencing (tNGS) has considerable potential for pathogen detection and distinguishing microbial profile differences between CAP and sCAP.

**Methods:**

In this retrospective cohort study, we analyzed 380 bronchoalveolar lavage fluid (BALF) samples from patients diagnosed with CAP or sCAP who underwent both conventional tests (CTs) and tNGS to identify and compare the pathogen species and clinical consistency.

**Results:**

The results were as follows: tNGS demonstrated a significantly higher clinical consistency rate with the final diagnosis (83.02%) than CTs (38.37%), with a statistically significant difference (*p* < 0.05). Among the analyses, the most prevalent combination of pathogen co-infection was mixed infection, with bacterial-viral co-infection being the most common, accounting for 40.7% of cases. Diverse microbial profiles were present in 72.94% (62/85) of patients with sCAP. Significant differences in the composition of diverse microbial profiles were observed between patients with CAP and those with sCAP (*p* = 0.001). Resistance genes were detected in 31 patients, of whom 35.48% (11/31) were consistent with CTs.

**Conclusion:**

Targeted next-generation sequencing demonstrated superior performance as a sensitive auxiliary diagnostic tool, which resulted in an increased pathogen identification rate and higher clinical diagnostic consistency. Although tNGS provides more comprehensive detection of etiological agents, clinical interpretation, diagnosis, and prediction require careful integration with conventional tests. The distinct clinical features, pathogen profiles, and pathogen composition between patients with CAP and those with sCAP highlight the need for a comprehensive diagnostic approach in pneumonia management.

## Introduction

Community-acquired pneumonia (CAP) is a widespread infectious disease that can lead to severe outcomes, which is caused by various pathogens, including bacteria, fungi, viruses, and atypical pathogens. The clinical management of pneumonia, along with long-term sequelae experienced by survivors of CAP and severe community-acquired pneumonia (sCAP), represents a substantial economic burden worldwide (Traber and Mizgerd, 2025). The clinical manifestations of pneumonia range from mild pneumonia, which presents with cough and fever, to severe pneumonia, which can progress to sepsis and acute respiratory failure (Aliberti et al., 2021). Pneumonia is typically diagnosed based on two or more symptoms, such as new-onset cough, temperature ≤36 °C or >38 °C, and dyspnea, and supported by chest computed tomography findings (Vaughn et al., 2024). The diagnosis of sCAP is evaluated based on the criteria of the *American Thoracic Society/Infectious Diseases Society of America Consensus Guidelines on the Management of Community-Acquired Pneumonia in Adults (ATS/IDSA Guidelines)* (Niederman and Torres, 2022). Physicians predict outcomes based on guidelines, prognostic scoring tools, and clinical course, and initiate mechanical ventilation, fluid resuscitation, and intensive care unit (ICU) critical care nursing when clinically indicated.

Overlapping comorbidities, low identification accuracy of infected pathogens, and overuse of broad-spectrum antibiotics result in multiple challenges in the diagnosis and management of pneumonia (Zhang et al., 2024; Ling et al., 2025). In a large-scale study involving 27,879 participants (aged 30–79 years), the China Kadoorie Biobank found that the average 30-day case fatality rate for patients was 2.4 deaths per 100 admissions (Hu et al., 2022). The risk factors of CAP are typically multifactorial, including age, potential pulmonary diseases, and history of smoking, which significantly influence the underlying causes and complications of CAP (Vaughn et al., 2024). Pathogenic infections should also be considered in addition to the aforementioned factors. As an acute lower respiratory infection commonly caused by diverse microbes, pneumonia requires prompt identification, which constitutes a critical early diagnosis for implementing effective management in the clinical pathway for pneumonia (Ling et al., 2025; Traber and Mizgerd, 2025). Culture serves as the fundamental tool for the diagnosis of pneumonia. Pathogens can be isolated and identified through cultivation, and isolated pathogens can be tested for susceptibility to provide critical information to facilitate diagnosis and treatment (Buchan et al., 2021). However, the cultivation method has been criticized for its time-consuming nature, typically requiring at least 48 h to derive identification and susceptibility test results (Buchan et al., 2021; Zhang et al., 2024). Other pathogens (such as viruses) require 3–5 days for successful cultivation (Gauthier et al., 2023). Conventional tests are not capable of identifying all pathogens, leading to the missed detection of causative pathogens and an inability to recognize mixed infections (Doualeh et al., 2022). Vaughn et al. (2024) proposed that pathogens could be identified in ≤40% of hospitalized patients. Consequently, the insufficiency of pathogen identification and the time-consuming nature of conventional tests highlight the limitations of detection methods. There is a trend toward adopting more effective methods as new tools to assist pneumonia diagnosis. Next-generation sequencing (NGS) is a comprehensive, rapid, and accurate molecular method for pathogen detection that can effectively identify a broad range of pathogens and concurrently detect drug resistance genes (Gauthier et al., 2023; Zhang et al., 2024; Li et al., 2025). NGS is compatible with a broad range of clinical specimens, such as blood, bronchoalveolar lavage fluid (BALF), cerebrospinal fluid (CSF), and sputum. Targeted next-generation sequencing is performed using targeted primers or probes for the construction of a panel that covers the majority of common clinical pathogens (Zhang et al., 2024). Compared with conventional tests (CTs), targeted next-generation sequencing (tNGS) is capable of detecting a greater number of pathogens and demonstrates higher accuracy and provides diagnostic value (Dai et al., 2025).

Although the analytical performance between tNGS and CTs has been compared in numerous studies, there are critical gaps in the assessment of the diagnostic consistency of both methods. This retrospective cohort study systematically evaluated and compared the detection performance of tNGS and CTs and the clinical diagnostic consistency of these methods to determine their clinical utility. Pathogen distribution and co-infection patterns in CAP and sCAP were identified using tNGS. This study aimed to determine the diagnostic value of tNGS as an auxiliary diagnostic tool for early diagnosis, especially in the clinical management of sCAP.

## Methods

### Case recruitment and sample collection

A retrospective single-center cohort study was conducted at the First Affiliated Hospital of Ningbo University (Precision Diagnosis and Treatment Center) from March 1, 2024 to March 1, 2025 with 398 patients with CAP or sCAP, aiming to characterize the pathogen distribution discrepancy in CAP and sCAP and compare the consistency of clinical diagnoses and the detection consistency of tNGS and CTs. Eligible patients presented with typical pneumonia symptoms, such as hypothermia (body temperature < 35 °C), fever (body temperature > 38 °C), leukopenia, and leukocytosis. Supportive findings on chest CT were also required for enrollment. CTs and tNGS were performed to evaluate the impact of infection patterns of multiple pathogens and the emergence of antimicrobial resistance. Patients enrolled in the study underwent simultaneous tNGS and CTs with antimicrobial susceptibility testing (AST) to identify causative pathogens and drug resistance profiles.

The exclusion criteria were as follows: (1) age < 18 years (n = 6); (2) tNGS reports unavailable prior to hospital discharge or death (n = 4); (3) tNGS analysis not performed on BALF samples (n = 2); (4) incomplete clinical records or baseline information (n = 2); and (5) lack of corresponding conventional culture or AST results (*n* = 4).

**Figure 1:**
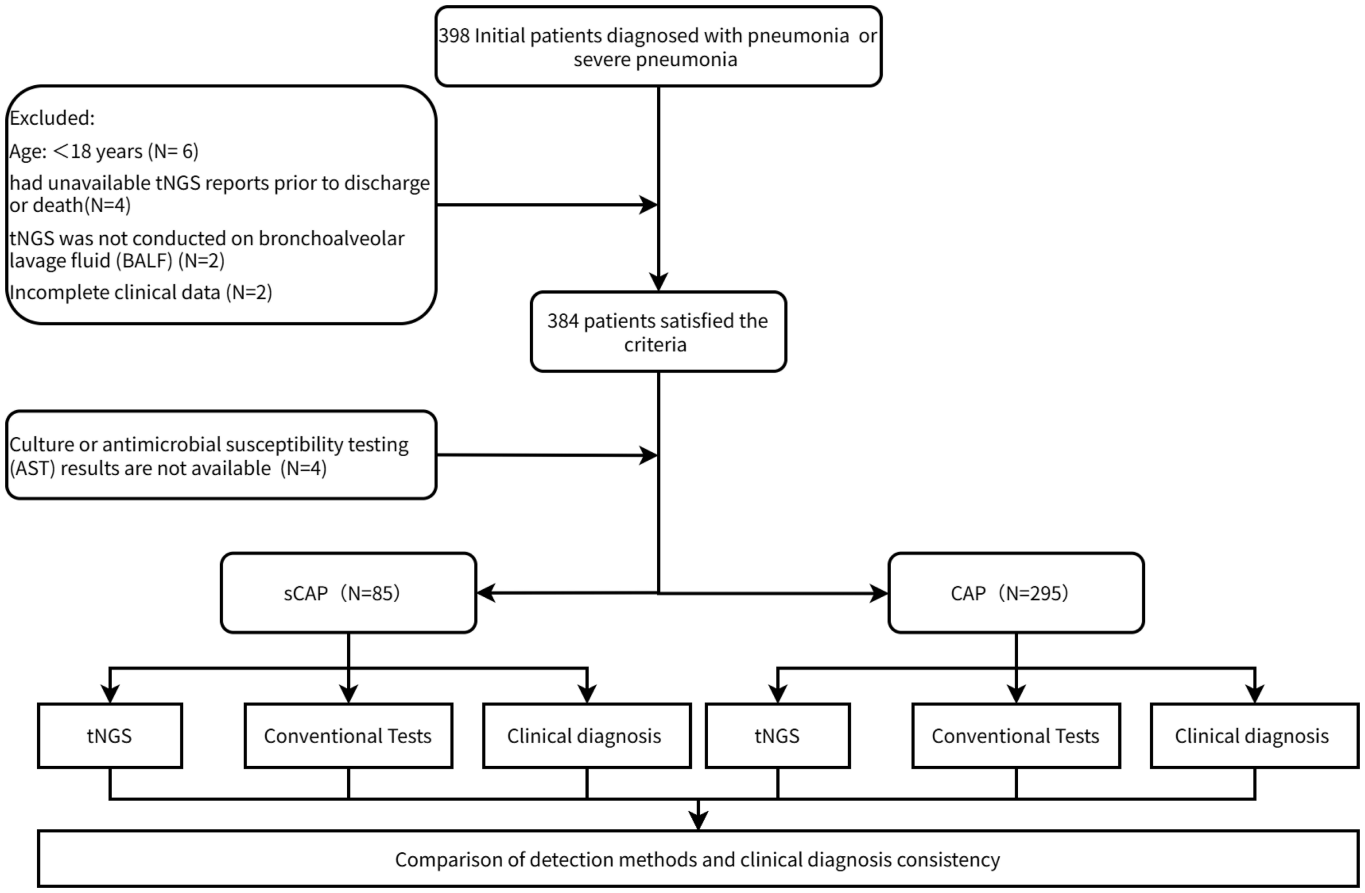
Overview of the experimental design and patient enrollment flowchart. Of 398 patients screened, 380 were enrolled (295 CAP, 85 sCAP) and underwent tNGS and conventional tests.

The patient enrollment flowchart is presented in Figure 1. After rigorous screening, 380 patients satisfied the criteria and were enrolled in the final analysis. Of these, 85 patients were classified into the sCAP group and 295 were classified into the CAP group based on the *Chinese Expert Consensus on Clinical Practice for Emergency Severe Pneumonia* and the *ATS/IDSA Guidelines on the Management of Community-Acquired Pneumonia in Adults* (Mandell et al., 2007; Society of Clinical Emergency Medicine, Chinese Medical Doctor Association, 2016). All participants underwent parallel testing using tNGS and conventional microbiological tests. Comparisons between these methods and clinical diagnoses were performed to evaluate detection consistency and diagnostic performance.

### Conventional microbiological tests and tNGS

Various conventional microbiological tests were used to identify pathogens, including Gram staining, bacterial culture, fungal culture, acid-fast bacilli staining, and antigen and antibody testing. For bacterial detection, BALF specimens were inoculated onto sheep blood agar, chocolate agar (incubated in 5% CO_2_ atmosphere), and MacConkey agar at 35–37 °C for 24–48 h. For fungal detection, samples were cultured on Sabouraud dextrose agar at 25–35 °C for up to 14 days. Gram staining was performed using a standard four-step procedure, and acid-fast bacilli staining was conducted using the Ziehl-Neelsen method. For viral pathogen detection, nasopharyngeal swabs were tested using a multiplex polymerase chain reaction (PCR) panel targeting six common respiratory pathogens (respiratory syncytial virus, adenovirus, rhinovirus, *Mycoplasma pneumoniae*, *Chlamydia pneumoniae*, and human bocavirus) or a multiplex PCR panel targeting 13 common respiratory pathogens (influenza A, influenza B, influenza A H1N1 subtype, influenza A H3N2 subtype, parainfluenza virus, rhinovirus, human metapneumovirus, coronavirus, respiratory syncytial virus, human bocavirus, adenovirus, *Mycoplasma pneumoniae*, and *Chlamydia pneumoniae*).

The GeneDian Targeted Gene Pathogen Detection Kit was used for tNGS analysis. Pathogenic nucleic acids were extracted from BALF samples and concentrated in accordance with the manufacturer’s instructions. Qualified nucleic acids were measured and recorded using a Qubit 4.0, and then enriched by targeted ultra-multiplex PCR amplification. Unique identification tags were added to the samples. Finally, DNA purification magnetic beads were added to remove excess adapters, dimers, and residual reagents, purifying high-quality targeted libraries for sequencing reactions. A product of library preparation with a concentration of ≥1 ng/μL, measured using a Qubit 4.0, was considered qualified. Based on the qualified library concentration, library pooling and DNA nanoball (DNB) preparation were performed. A DNB concentration of 8–40 ng/μL was considered qualified. An MGISEQ-200 gene sequencer was used to perform tNGS.

### tNGS bioinformatics analysis

Fastp (v 0.23.4) was employed to conduct a quality assessment of raw sequencing reads, remove adapter sequences, and trim low-quality bases. The preprocessed reads were aligned to the human reference genome (hg38) using bwa (v 0.7.17-r1188) to filter out human-derived sequences. The remaining reads were aligned to a custom-built tNGS pathogen database for species identification and classification. Samtools (v 1.6) was used to sort, remove duplicates, and index the SAM files generated by BWA, preparing the data for downstream analyses. Freebayes (v 1.3.6) was used to detect single-nucleotide polymorphisms (SNPs) and small insertions/deletions (indels) within the aligned reads, offering critical information on the genetic diversity and potential resistance mechanisms of the pathogens, including the identification of drug resistance mutations. Phylogenetic and lineage analyses of the detected pathogens, particularly for SARS-CoV-2 genomes, were performed using pangolin (v. 4.3) to rapidly assign phylogenetic lineages and provide valuable insights into their evolutionary relationships and transmission dynamics. Rscript (v. 3.5.1) was used to generate comprehensive statistical analyses and high-quality visualizations to facilitate the interpretation and presentation of the findings. *p* < 0.05 was considered statistically significant.

### Clinical diagnosis of CAP and sCAP

The diagnosis of CAP required the presence of typical clinical symptoms (e.g., fever, cough, leukocytosis, or leukopenia) along with supportive chest radiographic findings. sCAP was defined according to the *Chinese Expert Consensus on Clinical Practice for Emergency Severe Pneumonia* and *ATS/IDSA Guidelines on the Management of Community-Acquired Pneumonia in Adults* (Mandell et al., 2007; Society of Clinical Emergency Medicine, Chinese Medical Doctor Association, 2016), based on comprehensive clinical symptoms, including the following criteria:

Major criteria were as follows: (1) endotracheal intubation and invasive mechanical ventilation; and (2) septic shock requiring vasopressor therapy despite adequate fluid resuscitation. Minor criteria were as follows: (1) respiratory rate ≥ 30 breaths/min; (2) oxygenation index (PaO₂/FiO₂) ≤ 250 mmHg; (3) multilobar infiltrates; (4) altered consciousness and/or disorientation; (5) blood urea nitrogen ≥ 7.14 mmol/L; and (6) systolic blood pressure < 90 mmHg requiring adequate fluid resuscitation. Patients were classified as having sCAP if they met one or more major criteria (out of two) or three or more minor criteria (out of six).

### Concordance evaluation

CTs were performed in accordance with the guidance in the *Manual of Clinical Microbiology*. Quality control for CTs included the following: (1) specimen quality assessment, with sputum and BALF samples screened by Gram stain to ensure adequate cellular material and minimize oral contamination (accepted specimens had < 10 squamous epithelial cells per low-power field); (2) culture interpretation criteria, in which potential pathogens were distinguished from contaminants based on predominant growth, colony morphology, and clinical correlation, with common skin flora considered contaminants unless repeatedly isolated or clinically indicated; (3) confirmatory testing, in which presumptive pathogens were confirmed by biochemical identification, matrix-assisted laser desorption/ionization time-of-flight mass spectrometry and 16S ribosomal RNA gene amplicon sequencing.

tNGS was performed using a GeneDian Targeted Gene Pathogen Detection Kit. The kit is designed to identify bacteria (*n* = 160), fungi (*n* = 91), viruses (DNA viruses: n = 35, RNA viruses: *n* = 86), parasites (*n* = 21), special pathogens (*n* = 38), as well as antimicrobial resistance genes (*n* = 51), resistance loci (*n* = 50), and virulence genes (*n* = 32). Quality control for tNGS included the following: (1) criteria were established for pathogen identification, requiring clinical and radiological evidence consistent with active lower respiratory tract infection; (2) a background flora baseline was established according to the *Expert Consensus on the Application and Practice of Targeted Next-generation Sequencing in Infectious Diseases* (Society of Clinical Microbiology of China International Exchange and Promotion Association for Medical and Healthcare, 2024) recommendations, with pathogen-specific read count thresholds applied to minimize the influence of environmental and reagent-derived signals; (3) fungal pathogens detected by tNGS were interpreted in conjunction with clinical symptoms and radiological findings to establish causality rather than colonization; (4) common colonized pathogens such as *herpesviruses* detected by tNGS were considered significant only when associated with characteristic clinical manifestations of active infection; and (5) all tNGS results were interpreted in conjunction with independent microscopic examination (Gram stain) performed by separate laboratory personnel blinded to tNGS results to assess bacterial morphology and phagocytosis by leukocytes (evidence of active infection), ensuring objective assessment of microbial significance.

After rigorous quality control to exclude contamination, colonization, and false-positive signals, concordance between the tNGS and CTs detection results was evaluated by comparing the identified pathogens.

### Clinical consistency evaluation

A diagnostic team comprising a team leader and two team members, all with extensive clinical experience (>5 years in the respiratory department), independently performed the diagnostic evaluations. An integrated assessment synthesizing clinical symptoms, chest computed tomography findings, CTs, and tNGS reports was performed by the diagnostic team to determine whether the patients were infected and to identify the definitive etiological agent. Disagreements between team members were initially resolved through consensus discussions and consultation with another senior reviewer if agreement could not be reached. For diagnostically challenging cases that could not be resolved within the respiratory department, consultation with the infectious disease department was requested.

Patients with identified causative pathogens (e.g., invasive pulmonary aspergillosis and *Pneumocystis jirovecii* pneumonia) in the final clinical diagnosis documented in discharge summaries were included in the consistency analysis to ensure a robust evaluation.

Clinical consistency was assessed in patients with identified causative pathogens. Full consistency was defined as the detection of pathogens by tNGS/CTs that completely matched the clinical diagnosis. Partial consistency (no clinical impact) was defined as the detection of pathogens by tNGS/CTs that supported the clinical diagnosis, although additional pathogens were detected that did not interfere with the diagnostic conclusion. Partial consistency (with clinical impact) was defined as the detection of pathogens by tNGS/CTs that did not fully support the clinical diagnosis, in which major pathogenic agents identified in the clinical diagnosis were not detected by the respective method. Full inconsistency was defined as no overlap in the detected pathogens between the tNGS/CTs results and the clinical diagnosis.

The study protocol adhered to the principles of the Declaration of Helsinki and was reviewed and approved by the Medical Ethics Committee of the First Affiliated Hospital of Ningbo University (Precision Diagnosis and Treatment Center). Written informed consent was obtained from all participants or their legal guardians.

## Results

### Patients characteristics

Patients’ demographic information, comorbidities, predominant clinical symptoms, and prognoses are shown in Table 1. A total of 295 patients were diagnosed with community-acquired pneumonia, and 85 patients were diagnosed with severe community-acquired pneumonia. The study cohort included 215 males and 165 females, with a mean age of 62.00 [53.00, 72.00]. The prevalence of comorbidities was as follows: type 2 diabetes mellitus (*n* = 49), malignant tumors (*n* = 67), and non-infectious interstitial lung disease (*n* = 96). The predominant clinical symptoms were fever (*n* = 98), cough (*n* = 365), wet rales on lung auscultation (*n* = 118), leukocytosis (*n* = 98), and leukopenia (*n* = 24).

**Table 1:** Characteristics of 380 patients with pneumonia.

	Pneumonia	Severe pneumonia	*p* value
	(*n* = 295)	(n = 85)	
Sex (%)			0.001
Male	153 (51.9)	62 (72.9)	
Female	142 (48.1)	23 (27.1)	
Age (median [IQR])	60.00 [51.00, 69.50]	70.00 [61.00, 78.00]	<0.001
Fever status (%)			<0.001
Yes	63 (22.1)	35 (47.9)	
No	222 (77.9)	38 (52.1)	
History of malignant tumor (%)			0.002
No	253 (85.8)	60 (70.6)	
Yes	42 (14.2)	25 (29.4)	
Diabetes mellitus (%)			0.351
No	260 (88.1)	71 (83.5)	
Yes	35 (11.9)	14 (16.5)	
Lung tumor (%)			0.292
No	276 (93.6)	76 (89.4)	
Yes	19 (6.4)	9 (10.6)	
Non-infectious interstitial lung disease (%)			0.088
No	227 (76.9)	57 (67.1)	
Yes	68 (23.1)	28 (32.9)	
Discharge status (%)			<0.001
Improved	286 (96.9)	53 (62.4)	
Death	0 (0.0)	7 (8.2)	
Unimproved	0 (0.0)	11 (12.9)	
Others	0 (0.0)	2 (2.4)	
Missing	9 (3.1)	12 (14.1)	

IQR, Interquartile Range.

### sCAP and CAP infection pathogens distribution comparison

**Figure 2:**
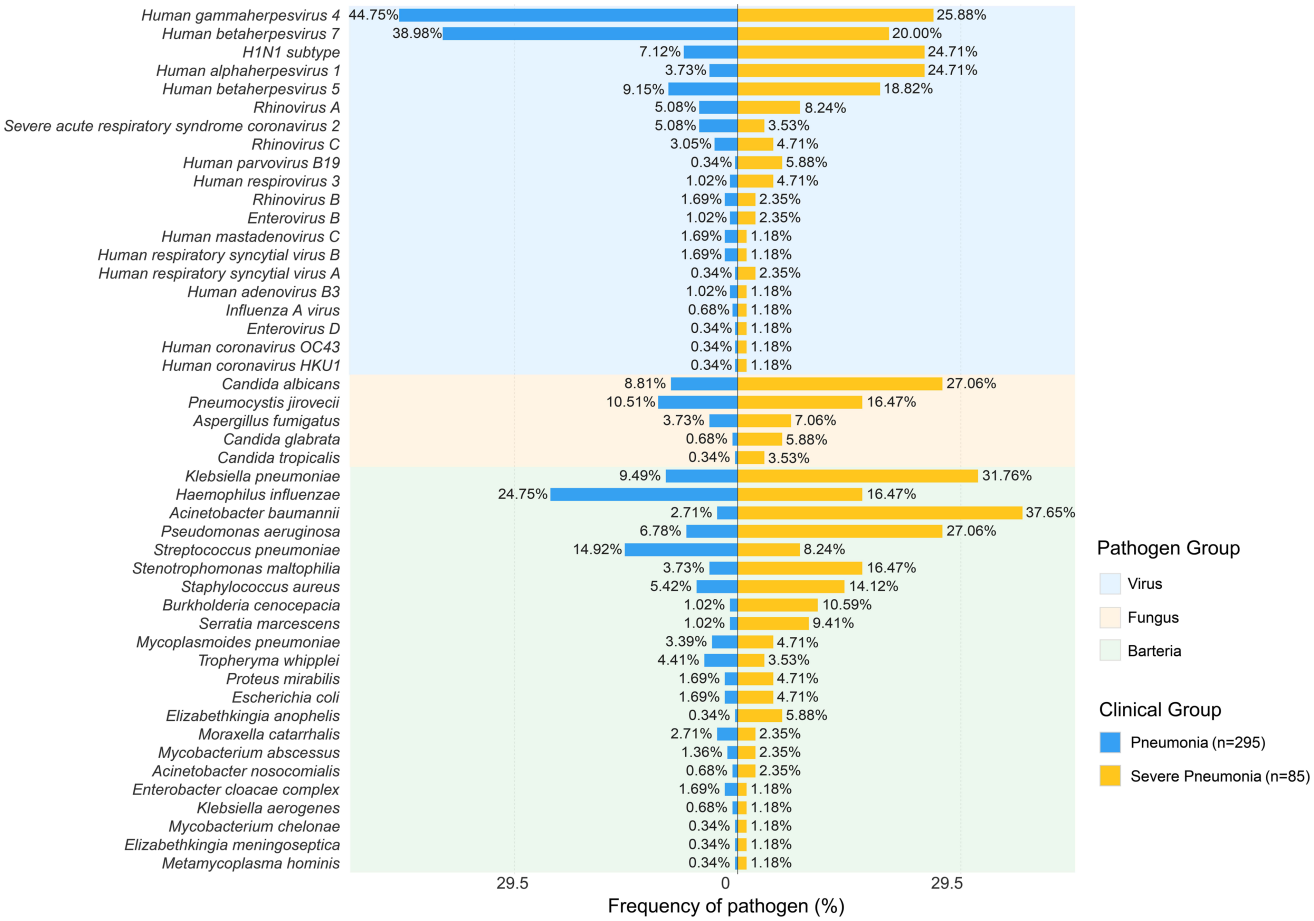
Frequency of pathogen in 380 samples. The comparison of pathogen detection frequency in CAP (left) and sCAP (right). Pathogens were divided into three groups: the virus group, the fungus group, and the bacteria group.

Based on the tNGS results, we categorized the pathogens into bacteria, fungi, and viruses. Across all patients, tNGS identified 86 distinct pathogenic species. Pathogen detection and distribution are shown in Figure 2. The top five most frequently identified pathogens were human gammaherpesvirus 4 (40.79%), human betaherpesvirus 7 (35.79%), *Haemophilus influenzae* (23.16%), *Klebsiella pneumoniae* (14.47%), and *Streptococcus pneumoniae* (14.21%).

The pathogen distribution differed significantly between patients with CAP and those with sCAP. Overall, infections in patients with sCAP were predominantly bacterial and fungal, whereas infections in patients with CAP were predominantly viral and bacterial. In the viral group, patients with CAP had a higher detection rate of human gammaherpesvirus 4 (44.75%) and human betaherpesvirus 7 (38.98%), whereas patients with sCAP had a higher detection rate of human gammaherpesvirus 4 (25.88%), human betaherpesvirus 7 (20.00%), influenza A H1N1 subtype (24.71%), human alphaherpesvirus 1 (24.71%), and human betaherpesvirus 5 (18.82%). In the fungal group, the pathogen detection results of patients with sCAP indicated a higher *Candida albicans* detection rate with 27.06% and *Pneumocystis jirovecii* detection rate with 16.47% than those of patients with CAP. In the bacterial group, pathogen detection in patients with CAP had a higher *Haemophilus influenzae* detection rate (24.75%) and *Streptococcus pneumoniae* detection rate (14.92%), whereas patients with sCAP had a higher detection rate of *Acinetobacter baumannii* (37.65%), *Klebsiella pneumoniae* (31.76%), and *Pseudomonas aeruginosa* (27.06%).

### Pathogens infection patterns

**Figure 3:**
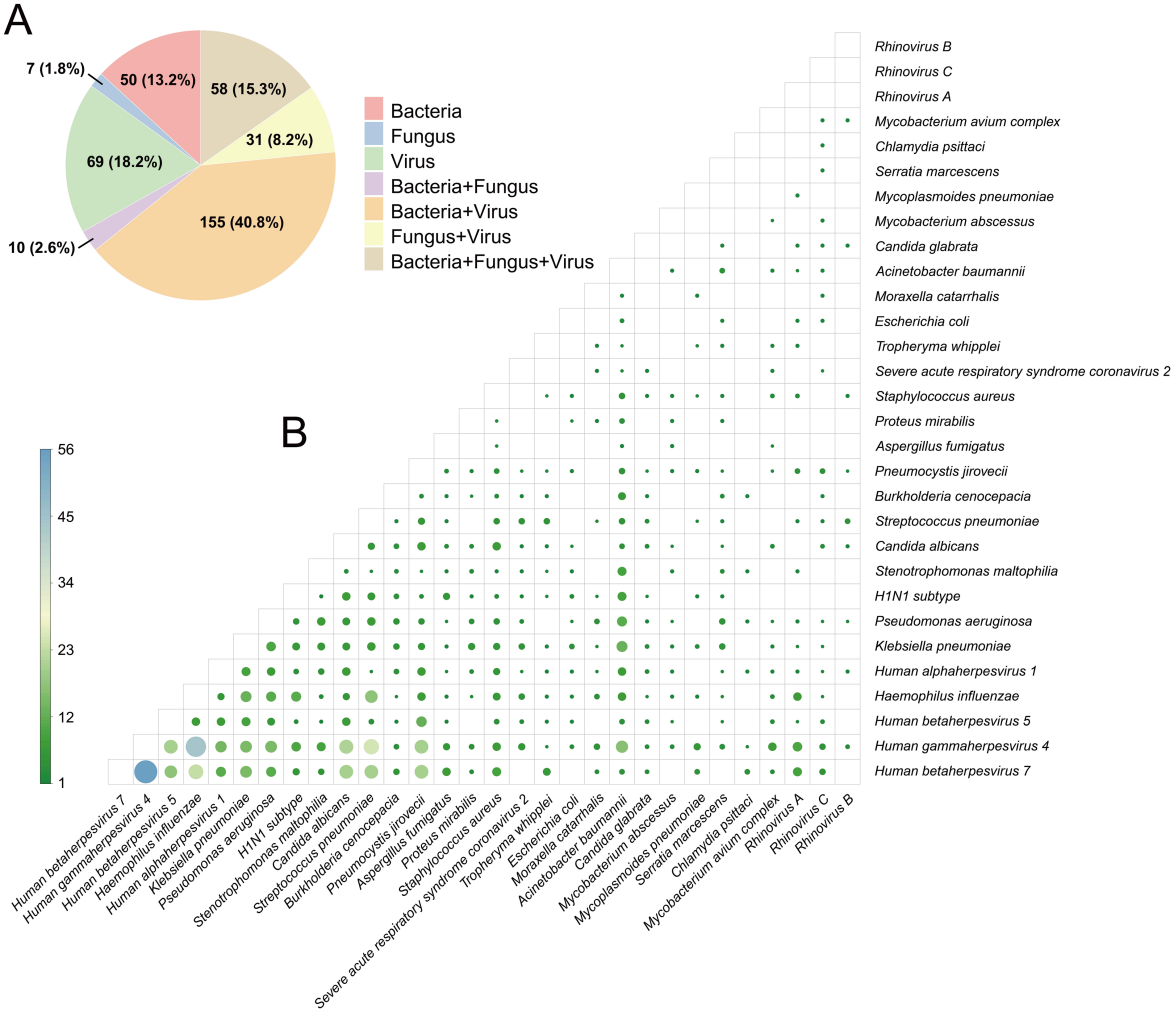
Mixed infection pattern in 380 samples. **(A)** Infection patterns in 380 samples. **(B)** Mixed infection frequency of the top 30 pathogens. Each dot represents a pathogen pair. Darker colors and larger dot sizes correspond to higher mixed infection frequencies between the combination of two pathogens.

Additionally, the most predominant infection pattern was mixed infection, which occurred in 67% of the patients (*n* = 254/380), and 33% (*n* = 126/380) of the patients had a single infection. Based on the co-infection status, we further distinguished the infection patterns as bacterial-fungal co-infections, bacterial-viral co-infections, fungal-viral co-infections, and triple co-infections involving bacteria, fungi, and viruses. The tNGS detection results indicated that the combination of bacteria and viruses exhibited the highest co-infection rate of 40.7% (*n* = 155/380) (Figure 3A).

The detection frequency of mixed infections is shown in Figure 3B. The most frequently detected combination of pathogens was human gammaherpesvirus 4 and human betaherpesvirus 7. The second most frequent combination was *Haemophilus influenzae* and human gammaherpesvirus 4.

### Concordance evaluation between tNGS and CTs

**Figure 4:**
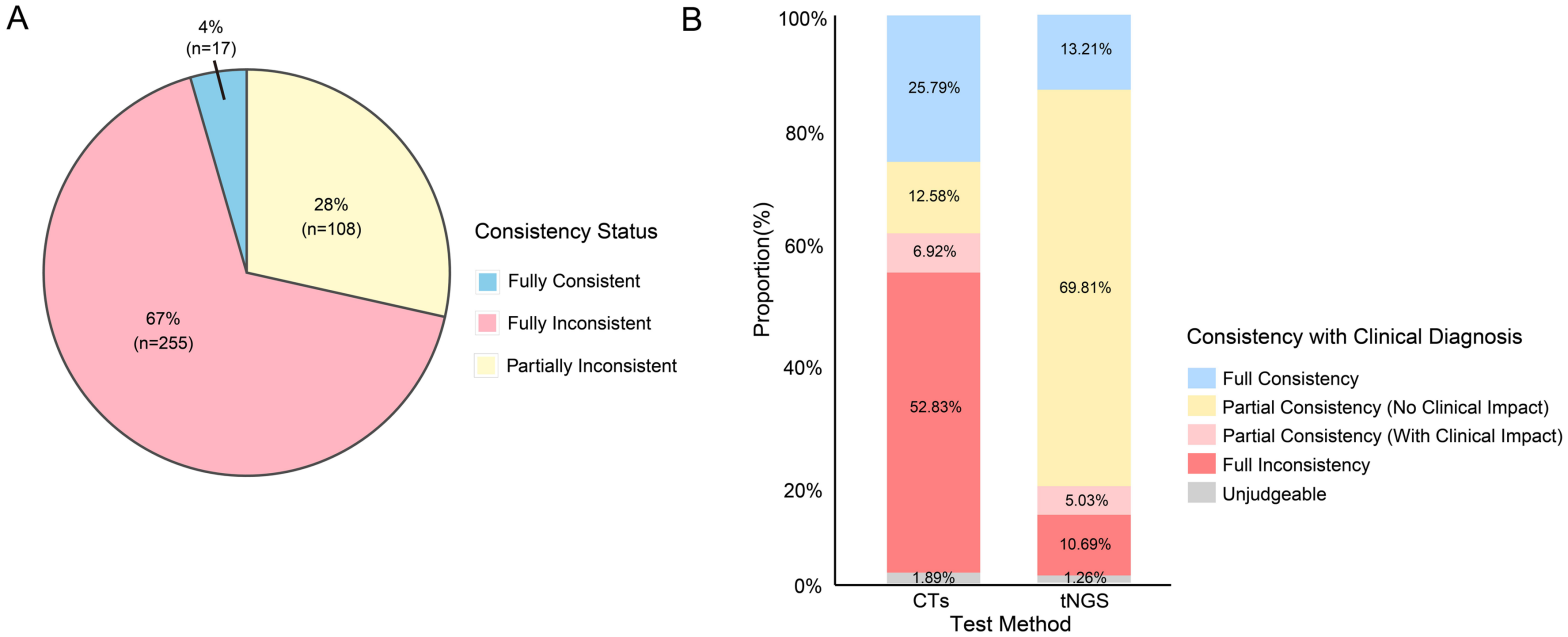
Consistency comparison. **(A)** Consistency comparison between tNGS and CTs. **(B)** Consistency comparison with clinical diagnosis.

A significant difference was observed in the concordance evaluation between tNGS and CTs. The comparison results showed that in 380 samples, 4% (*n* = 17) of the detection results were fully consistent between tNGS and CTs, 28% (*n* = 108) were partially consistent, and 67% (*n* = 254) were fully inconsistent (Figure 4A).

Resistance genes were detected in 31 patients, comprising eight distinct gene types. The primary pathogens included *Acinetobacter baumannii, Proteus mirabilis, Serratia marcescens*, and *Staphylococcus aureus*. The most frequently detected genes were *blaOXA*-23-like and *blaOXA*-51-like, which are primarily associated with carbapenem/penicillin resistance in *Acinetobacter baumannii*. In 11 patients, the resistance gene detection results were concordant with the phenotypic AST results.

### Diagnostic performance comparison

We evaluated the clinical consistency of tNGS and CTs in terms of diagnostic performance. Based on a comprehensive assessment of patients’ clinical presentation, disease progression, conventional testing results, and tNGS results, 159 patients were diagnosed with a definitive pathogenic diagnosis. In this group, the comparison between the tNGS detection results and clinical diagnoses showed that 13.21% of the samples showed full consistency, 69.81% showed partial consistency (no clinical impact), 5.03% showed partial consistency (with clinical impact), and 10.69% showed full inconsistency. The comparison between the CTs results and clinical diagnoses showed that 25.79% showed full consistency, 12.58% showed partial consistency (no clinical impact), 6.92% showed partial consistency (with clinical impact), and 52.83% showed full inconsistency (Figure 4B).

## Discussion

The detection rate of tNGS was notably higher than that of CTs, aligning with the findings of numerous previous studies (Ma et al., 2024; Dai et al., 2025; Xu Q. et al., 2025). The results demonstrated that tNGS has a definite advantage over CTs for pathogen detection, especially for rare pathogens. In comparison with clinical diagnoses, among patients with CAP, especially those with sCAP, microbial infections caused by infectious pathogens play a significant role in contributing to acute respiratory distress syndrome (ARDS) or severe acute lung injury, which critically influences the management of pneumonia (Lee, 2017). The tNGS panel employed in this study was designed to target 431 common clinical pathogens. Through the evaluation of clinical diagnostic consistency, the results confirmed that there were no unusual or unique pathogens beyond the analytical capability and detection range of tNGS. In this study, tNGS exhibited a superior detection rate, average detection time, and clinical diagnostic concordance.

Human gammaherpesvirus 4, commonly known as *Epstein–Barr virus* (EBV), is broadly distributed in approximately 90% of adults worldwide (Liang et al., 2023), and is the most frequently detected pathogen in this research. The detection of active EBV infection in the healthy population is rare because the virus remains in a latent state in most individuals, while its prevalence increases progressively with human age (Zhao et al., 2024). The estimated prevalence rate of human herpesvirus 7 (HHV-7) was 44% in previous large-scale data reported during the coronavirus disease (COVID-19) pandemic (Shafiee et al., 2023). The detection results were in accordance with those from previous large-scale data.

The most frequently detected combinations of pathogens in this study were EBV and HHV-7. The presence of EBV and HHV-7 does not indicate active pneumonia etiology in its latent state as a colonizing pathogen. However, the reactivation rates of EBV and HHV-7 increased progressively with age, considering the mean age of 62.0 in this study, pneumonia caused by viral infections deserves attention for the prevalence of immunosenescence or comorbid illness.

An analysis of the distribution of pathogens in 380 patients indicated that the dominant pathogens detected in CAP differed from those detected in sCAP. The dominant infection pattern in both CAP and sCAP was mixed infection, accounting for 67% of all patients. In this study, the mixed infection rate was notably higher than the proportion of mixed infections in a national study conducted by Liu et al., from 2009 to 2020 across 30 provinces in China (Liu et al., 2023). However, the co-infection trends were similar, indicating that bacterial-viral co-infections were the dominant clinical co-infection patterns (Liu et al., 2023; Xu Z. et al., 2025). This discrepancy was assumed because the national study conducted by Liu et al., mainly adopted CTs to detect pre-assumed pathogens, thereby missing co-infections involving pathogens outside the detection range. Thus, in our study, mixed detection was observed at a higher proportion. This discrepancy highlights that tNGS results can identify more pathogenic and opportunistic pathogens that could result in infection. Numerous prior studies have indicated that bacterial-viral co-infections could result in impaired presentation because synergistic effects could enhance the severity of pneumonia (Voiriot et al., 2016; Manna et al., 2022). Adults with sCAP have been found to be significantly associated with infection or co-infection with *Pseudomonas aeruginosa, Klebsiella pneumoniae,* and *Streptococcus pneumoniae* in previous studies (Liu et al., 2023), which is consistent with the distribution of the bacterial pathogens detected in this study. Owing to the limitations of conventional tests, the detection of viral infections could be lower because of the difficulty of viral culture and the inadequate assumption of PCR (Huang et al., 2020). This phenomenon is evidenced by the substantial increase in the viral infection rate in patients with CAP in recent years (Shang et al., 2020), driven by the application of NGS technology. This implies that CTs may have missed a significant number of cases of viral co-infection. Prior research indicates that in elderly patients, underlying conditions could be exacerbated by viral infection (Falsey, 2007). Thus, to recognize pathogens detected by tNGS as pathogenic or colonized, a comprehensive consideration of the patients’ clinical indicators and symptoms to guide appropriate antimicrobial therapy is an opportunity and challenge for tNGS.

A comparison of detection consistency between tNGS and CTs revealed that 4% was full consistency, 28% was partial consistency, and 67% was full inconsistency. The difference in proportion was determined by the detection discrepancy between tNGS and CTs. Conventional microbiological tests include microbial culture, enzyme immunoassay, polymerase chain reaction (PCR), and a combination of these methods (Lin et al., 2023; Chen et al., 2025). CTs are fundamentally limited by their technical limitations, including the inability to cultivate non-viable bacteria, interference of culture results by the initial bacterial load in a sample, and failure of fastidious bacteria to grow within the standard culture time. In contrast, tNGS is less affected by these factors and can detect a broad spectrum of pathogens by leveraging enrichment techniques and lowering the detection limit. However, tNGS also has several limitations. For instance, the tNGS detection result of a sample collected during the early stages of the disease may not be able to cover all pathogens in the final diagnosis because patients could contract hospital-acquired infections during the later period of hospitalization.

In earlier diagnoses, tNGS could detect more pathogens than CTs, and the study indicated better concordance with the clinical diagnoses. The results indicated that the consistency between tNGS and clinical diagnoses was significantly higher than that between CTs and clinical diagnoses (83.02% vs. 38.37%, *p* < 0.05), which is consistent with prior research (Dai et al., 2025). The comparison of clinical diagnosis consistency was the summation of the proportions of full and partial consistency (no clinical impact). In this retrospective study, only a limited number of pathogens could be identified by culture. The enrichment process in tNGS improves the nucleic acid concentration of pathogens, allowing tNGS to identify more pathogens that conventional tests cannot detect (Zhang et al., 2024). Three clinicians evaluated the clinical diagnoses, and only the main pathogenic nosogenesis was recorded. Thus, for full clinical diagnosis consistency proportion comparison, CTs were slightly higher than tNGS because colonizing bacteria and lower loads of pathogens detected by tNGS were not considered pathogenic, whereas partial clinical diagnosis consistency (no clinical impact) in tNGS exhibited a significantly higher proportion than CTs because the limitations of CTs resulted in the failure to detect pathogenic pathogens.

This comparison indicates higher clinical consistency of tNGS than that of CTs, because tNGS usually detects more pathogens than CTs. However, the selection of detection methods requires balancing multiple factors. Detection breadth varies substantially by panel design, with tNGS panels typically ranging from hundreds to thousands of predefined targets, in contrast to metagenomic next-generation sequencing (mNGS), with its theoretically unbiased coverage and PCR’s narrower target-specific approach. Thus, cost and turnaround time also significantly influence clinical decision-making. Common clinical methods used for pathogen identification include CTs (including molecular diagnostic techniques such as PCR), multiplex PCR-based tNGS (mp-tNGS), hybrid capture-based tNGS (hc-tNGS), and mNGS.

Although this study compared the detection performance between mp-tNGS and CTs, the existing literature suggests comparable diagnostic accuracies between mNGS and tNGS approaches (Yin et al., 2024; Yang et al., 2025). Specifically, mp-tNGS and hc-tNGS achieved sensitivities of 86.5 and 87.3%, and specificities of 90.0 and 88.0%, respectively, which is comparable to those of mNGS (85.5% sensitivity, 92.1% specificity) (Yin et al., 2024).

The clinical adoption of these technologies also depends on their economic feasibility. In China, mNGS (DNA procedure only) currently costs ¥3,000–3,500 ($420–500) per specimen (Yin et al., 2024; Wang and Zhao, 2025), whereas mp-tNGS and hc-tNGS are priced at roughly a quarter to half of mNGS, with mp-tNGS ~$120 per specimen and hc-tNGS ~$300 per specimen (Yin et al., 2024). CTs remain the most economical diagnostic options; for example, PCR costs approximately $5–10 per sample for single assays, substantially lower than tNGS or mNGS (Osei Sekyere, 2025). However, as CTs require multiple sequential tests and have a limited detection range, comprehensive pathogen detection methods may offer cost-effectiveness per specimen.

In terms of turnaround time, conventional culture requires 3–7 days for general pathogen identification, whereas mNGS typically requires 40–51 h (Wang and Zhao, 2025), mp-tNGS requires ~ 10 h, and hc-tNGS requires 16–23 h (Yin et al., 2024). PCR-based methods offer the fastest detection, with a single assay delivering results within 1–2 h (Osei Sekyere, 2025).

Therefore, an integrated diagnostic approach that combines tNGS and CTs should be considered to provide patients with rapid and comprehensive pathogen identification at different stages of the disease.

This study used tNGS to conduct an in-depth analysis of microbial profiles in the lower respiratory tract, revealing significant differences between patients with CAP and those with sCAP. BALF research has provided crucial insights into the microbial environment of the lower respiratory tract. However, several limitations of this study warrant consideration, most notably the potential for selection bias arising from the exclusion of patients with incomplete clinical records or unavailable tNGS reports. Although exclusions due to loss to follow-up or quality control failures could theoretically skew results, we believe that our final cohort (*n* = 380) remains highly representative of real-world clinical practice. To this end, a sensitivity analysis revealed no statistically significant differences in baseline demographics (except for age) or clinical presentations between the included and excluded patients (all *p* > 0.05 for indicators other than age), suggesting that the exclusion process was largely random rather than systematic. Moreover, as our cohort (*n* = 380) encompasses a broad spectrum of pneumonia severities (CAP vs. sCAP) and diverse pathogen profiles, these findings offer robust insights into real-world diagnostic performance. Nevertheless, the small sample size and single-centre design represent limitations that should be addressed in future multi-centre trials. Future prospective studies with consecutive enrollment are warranted to further minimize potential bias and ensure broader generalizability across diverse clinical settings.

## Conclusion

In conclusion, this study revealed significant differences in clinical features and microbial profiles between patients with CAP and sCAP, highlighting the importance of comprehensive diagnostic methods. Compared with CTs, tNGS exhibited a higher detection rate, higher diagnostic consistency, and better efficiency, indicating significant clinical value. As a more sensitive method for pathogen detection, especially in sCAP, tNGS has comprehensive diagnostic value. Despite its broad detection capability, tNGS cannot replace CTs in clinical diagnosis. The detection results of tNGS require urgent standardization in interpretation to minimize variability arising from differences in the expertise of medical professionals. Therefore, an integrated treatment that combines tNGS and CTs is recommended for optimal patient management. Treatment medications should be adjusted based on comprehensive diagnostic results.

## Data Availability

The data presented in the study are deposited in the NCBI with accession number: PRJNA1462495.
